# What do clinicians perceive as the effective implementation strategies for TREAT journal clubs? A qualitative study

**DOI:** 10.1186/s12909-025-06929-x

**Published:** 2025-03-24

**Authors:** Rachel Wenke, Jodie Wiseman, Paulina Stehlik, Caitlin Brandenburg, Katherine Richards, Sharon Mickan

**Affiliations:** 1 Gold Coast Health, Southport, Australia; 2 Bond University, Gold Coast, Australia; 3 Griffith University, Gold Coast, Australia; 4 University of the Sunshine Coast, Sunshine Coast, Australia

**Keywords:** Journal club, Evidence-based practice, Allied health, Sustainability

## Abstract

**Background:**

Journal clubs have long been recognised as a potential tool for supporting evidence-based practice skills and culture, however they can be challenging to implement and sustain in clinical settings. While the TREAT journal club format was developed to address some of these challenges; it is unclear which strategies are most helpful in supporting ongoing sustainability. To further this enquiry, the aim of this study was to identify clinician’s perspectives of the most effective implementation strategies for supporting sustainable TREAT journal club attendance, culture and satisfaction.

**Methods:**

Clinicians, clinician-facilitators, and research mentors were recruited from six allied health journal clubs who participated in the TREAT journal club format within a single hospital and health service. Participants were invited to attend focus groups at 10 months and 16-months following participation in their journal club. Focus group questions explored which strategies participants felt were most effective during implementation of the journal club, what outcomes they led to and what if any contextual factors influenced these outcomes. Data analysis involved an inductive and deductive approach and the formation of context- mechanism-outcome configurations drawing from elements of a realist evaluation.

**Results:**

Eighteen focus groups were conducted separately with 47 clinician participants, 12 clinician-facilitators, and 6 research mentors. Strategies reported to be the most effective related to clinical relevance and application of the topic, group participation (i.e., group prioritisation of topics, group discussion), consistency, structure (i.e., protected time, structured appraisal tool, timetabling) and mentoring. These were further synthesised within 11 context-mechanism-outcome configurations which identified contexts that influenced outcomes. For example, clinicians reported smaller and single profession journal clubs enhanced outcomes relating to attendance, culture and practice changes when implementing the strategy of discussing the article’s application to practice.

**Conclusion:**

Clinicians report several strategies which may enhance journal club attendance and satisfaction, EBP culture, and knowledge and skills of clinicians when implementing a TREAT journal club for up to 16-months. Findings emphasise the importance of journal club topics being identified and prioritised by clinicians as a group to maximise relevance and clinician buy-in. Strategies discussed may be useful for services to consider when implementing journal clubs, taking into consideration specific contexts.

**Supplementary Information:**

The online version contains supplementary material available at 10.1186/s12909-025-06929-x.

## Background

Evidence Based Practice, the act of integrating findings from high quality evidence with patient preference and clinical knowledge, is a cornerstone of modern healthcare [1]. Journal clubs (JCs) are commonly used by health professionals as a means for integrating evidence into everyday clinical practice [2]. Journal clubs have been associated with improvements in evidence-based practice knowledge, research culture, critical appraisal skills, and importantly, influence or changes in clinical practice [3–5]. However, maintaining clinician interest and participation can be difficult, contributing to variable effectiveness in terms of influencing skills and practice and reduced sustainability [6].

The “Tailoring Research Evidence and Theory” or ‘TREAT’ journal club format [4] was developed to address some of these challenges and promote sustainability in clinician-led journal clubs. The TREAT format incorporates effective, evidence-based concepts for journal clubs including prioritising clinically relevant topics and incorporating principles of adult learning [7, 8]. A cluster randomised-controlled trial evaluating the TREAT format compared to traditional unstructured JC formats was conducted and identified significantly greater satisfaction with the TREAT format across a 6-month period [4]. No significant difference in EBP skills was identified; likely due to the short time frame of the trial [9]. 

Participants in the TREAT arm of this trial trial were interviewed to explore the sustainability of TREAT journal clubs following the 6-month implementation period with participants identifying several implementation strategies [10]. These strategies were then explored in a follow up implementation study which involved 132 unique participants from seven allied health professions applying the strategies as part of a tailored implementation plan to implement TREAT journal clubs [4]. Clinicians demonstrated significantly improved EBP skills and self-reported confidence at 10 and 16-months [4]. In addition, 88 of the clinicians reported adopting new treatments/resources and 64 reported updating clinical procedures as a result of the journal club. Most participants recommended the format and wanted to continue using it with the majority of the journal clubs being sustained for 16-months [4]. While brief survey responses revealed enablers and barriers to journal club implementation, it is still unclear which of these strategies were most effective in supporting longer term implementation.

Considering the widespread practice of journal clubs [11] and associated investment in time and resources into administering journal clubs internationally, it is important to identify effective strategies to support ongoing implementation and sustainability. Further exploration into strategies which support health professional’s attendance, culture and satisfaction with the journal club is required to continue to influence their sustainability [10]. The TREAT journal club format is a complex intervention, and the effectiveness of associated implementation strategies is influenced by many factors likely leading to different outcomes in different contexts. In order to consider which strategies are most effective in supporting longer term implementation of TREAT journal clubs, it is helpful to consider “how, why, for whom, to what extent, and in what context” (p.2) these strategies work [12]. Drawing from elements of a realist evaluation approach may be useful in addressing these considerations to understand which implementation strategies work, for whom and why [12]. It may be particularly helpful to understand which strategies influence outcomes previously reported to influence longer term journal club implementation or sustainability including journal club attendance, satisfaction and, evidence-based practice culture [10]. Greater insight into contextual factors that influence outcomes will also assist in choosing successful implementation strategies in different organisational contexts.

### Objectives

The primary aim of this study was to identify clinician’s perspectives of the most effective implementation strategies for promoting allied health professionals’ TREAT journal club attendance, satisfaction, and evidence-based practice culture across a 16-month period. The second aim was to deepen understanding of implementation strategies by identifying clinician’s perceptions of important contextual factors which influenced these specific outcomes.

## Methods

### Study design

#### Methodological orientation

The study formed part of a larger mixed methods evaluation which was guided by the Knowledge To Action Framework (KTA) [13]. This component employed a qualitative design, drawing from elements of a realist-informed approach. Specifically, the study focussed on practical implementation strategies and their observed outcomes rather than fully unpacking the causal mechanisms underlying these strategies. While the study uses elements of realist evaluation, including CMO configurations, it does not aim to provide a comprehensive exploration of generative mechanisms as per Pawson and Tilley’s framework [14]. Data was collected using focus groups with journal club participants. Results are reported in line with the COREQ reporting guidelines [15].

#### Setting

Participants were recruited from six participating allied health journal clubs within a large Australian metropolitan tertiary hospital and health service with an allied health workforce of approximately 1200 employees. Focus groups were completed at the health service site in person or via teleconference. All participants provided written informed consent and Human Research Ethics Committee Approval was received (HREC/16/QGC96) and the study adhered to the Declaration of Helsinki.

### Participants

Three participant groups were recruited:

1. Clinician participants: Clinician participants were considered eligible if they were an allied health professional who had participated in a TREAT journal club within the health service’s implementation study [4].
2. Clinician-facilitator participants: Allied health professionals who identified as a clinician facilitator in a TREAT journal club within the health service’s implementation study who had undergone targeted evidence-based practice training and had facilitated or co-facilitated TREAT journal club sessions.
3. Research mentors: Allied health professionals with a research qualification (PhD qualified) who received training to support TREAT journal club clinician-facilitators.

#### Participant selection

All eligible journal club members were emailed a focus group invitation, with an attached participant information and consent form and asked to contact the researcher if they were interested in attending. While all clinician facilitators who indicated interest were recruited, clinician participants were selected based on who responded first indicating their availability. For clinician participants, we used purposive sampling to ensure a spread of clinical experience (e.g., ranging from entry level, senior and team leaders), recruiting potential participants who had responded from a range of clinical experience levels. There was one exception for one journal club where the team leader had requested that all journal club members attend the focus group.

#### Journal club intervention

All participants had participated in at least one TREAT journal club session in the 16-month implementation period. The TREAT JCs were planned to run for one hour, once a month over approximately 16-months, allowing for flexibility with scheduling over holiday and clinically demanding periods. Details regarding the TREAT format can be found at www.treatjournalclubs.com however key components included: set roles during the journal club (i.e., facilitator, presenter and scribe), group-based appraisal of articles using the Critical Appraisals Skills Programme (CASP) structured appraisal tools [16] and group discussion of the evidence in the context of clinical practice. An example session plan is found in Supplementary File 1 with further details published elsewhere [5]. At least two clinician-facilitators were assigned to each journal club. Clinician-facilitators attended a local evidence-based practice workshop targeting critical appraisal skills and received a written guide and one on one training on delivering the TREAT format by a member of the research team. In addition, each journal club was supported by an allocated research mentor. The first two TREAT JC sessions were facilitated by the research mentor, with gradual co-facilitation and then independent facilitation by a clinician-facilitators as they felt confident.

Prior to the first JC session, journal club participants gave feedback about barriers to their journal club through a Journal Club Culture Questionnaire (see [4] for more details), after which the research mentor met with the clinician-facilitators and co-developed a tailored implementation plan based on this feedback. The plan included co-designed strategies which aimed to address barriers related to journal club participation and sustainability, with a number of strategies grounded on previous research and informed by the COM-B model of behaviour change, either addressing capability, motivation or opportunity [15].

#### Data collection

Focus groups were conducted with clinicians and clinician-facilitators at 10- and 16-months post journal club implementation. Two focus groups were also completed with research mentors at approximately 10 months post journal club implementation. Focus groups were facilitated by allied health professionals who were not directly involved with the respective clinical teams but had training in undertaking qualitative interviews. The focus group facilitators were encouraged to reflect after each interview on how their perspectives and experiences may have influenced their facilitation of each interview and had the opportunity to debrief with a team member after each focus group. The focus groups were semi-structured, aiming to explore participants’ experiences with participating in a journal club using the TREAT format. As detailed in Supplementary file 2, questions included discussing the most effective implementation strategies and what outcomes these strategies (if any) were associated with and what contexts (if any) may have impacted these outcomes. Specifically, participants were shown a list of the implementation strategies they had applied as part of their tailored implementation plan and asked to describe what they perceived to be the two to three most effective strategies. More general questions relating to factors influencing journal club attendance and satisfaction and evidence-based practice culture were also discussed. Focus groups varied in length, from approximately 30 min to 60 min, largely depending on the number of participants. Focus groups were audio recorded and transcribed verbatim using a professional transcription service.

#### Data analysis

Data was analysed using NVivo 12 software. Coding of data occurred in two phases; 12-month focus group data and 16-month focus group data. Initial coding of the 12-month data was completed by one investigator (JW) and checked by a second investigator (RW) incorporating a combined inductive approach using thematic analyses as described in Braun and Clarke [16] and deductive approach based on the mechanism, context and outcome elements of a realist approach. For example, each implementation strategy was initially coded as a mechanism, and associated outcomes and contexts were coded in relation to these mechanisms. Further data outside these mechanisms including future suggestions and other factors influencing outcomes were coded inductively. Effective mechanisms reported by participants across more than one journal club were reported and categorised into broad explanatory themes. Coding for 16-month focus group data was undertaken by RW and used a predominately deductive approach focussing on looking for relationships between the most effective implementation strategies reported, and associated outcomes and contexts. Factors which influenced attendance, satisfaction and EBP culture were also coded deductively against the categories obtained in the 10-month data. This data was checked by a second researcher (JW). Disagreements or uncertainties in coding were resolved via discussion between coders. Following this process, Context- Mechanism-Outcome (CMO) configurations were generated from the data. Although the authors originally conceptualised strategies as mechanisms, it was decided that for the purpose of CMO configurations, strategies would be labelled “strategy” which “enabled” mechanisms. Mechanisms were pragmatically conceptualised by the lead author (RW) from review of the data as the processes that caused outcomes and reviewed and refined by the wider team. All results were presented to the group with opportunity for discussion and refinement. Throughout the research, authors also reflected on how their own subjectivities may have influenced their interpretation of the results and endeavoured to put aside any potential biases. The team of six authors varied in their experience with the TREAT format as well clinical and research experience and included experts in qualitative research, knowledge translation, clinical education and health management. The varied experiences allowed for a balanced methodological approach and robust debate and discussions within the team.

## Results

### Participants

A total of 132 participants were eligible to participate in focus groups. Using purposive sampling, 84 participants across six journal clubs were invited to participate across the two time-points (see Table 1). Reasons for nonattendance were predominately related to unavailability. Eighteen focus groups were conducted, including 11 focus groups at 10 months and 7 focus groups at 16-months with 47 clinician participants and 12 clinician-facilitators (4 clinicians and 5 clinician-facilitators completed focus groups across both time points). Most participants were from Occupational Therapy (35%), Speech Pathology (26%) or Oral Health (26%) with representation also from Pharmacy (6%), Dietetics (6%) and Physiotherapy (2%). Complete details of participant demographics of these journal clubs have been previously published [4].

**Table 1:** Participant numbers

Journal club name	Clinicians 10 -months	Clinician-Facilitators 10-months	Research mentors 10-months	Clinicians 16-months	Facilitators 16-months
Journal club 1	1	1	1	Not completed	2
Journal club 2	5*	2	1	Not completed	
Journal club 3	14	Not completed	1	Not completed	
Journal club 4	7	4	1	7	2
Journal club 5	3	1	1	4	1
Journal club 6	2	2	1	4	2
**TOTAL**	**32**	**10**	**6**	**15**	**7**

^*^Note this focus group included 1 single interview

Six research mentors, an average of 7.6 years post PhD (range = 4 to 17 years), also participated in one of two focus groups approximately 10 months post journal club implementation.

### Most effective implementation strategies

**Fig. 1:**
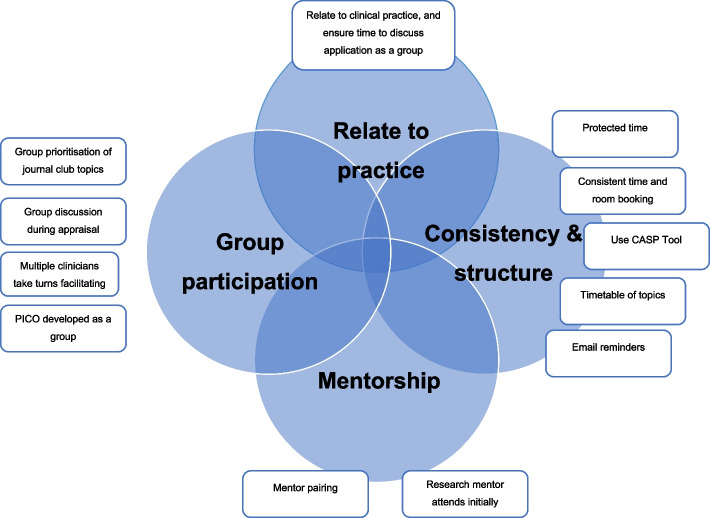
Key strategies for effectively implementing a TREAT journal club

Clinicians in each journal club had the opportunity to describe which implementation strategies they felt were most effective. The most prevalently identified strategies were synthesised into four key themes as depicted in Fig. 1. These strategies are further outlined in Table 2 together with the number of unique journal clubs reporting them as most effective and the outcomes associated, which will now be described together with influencing contexts.

**Table 2:** Strategies identified as most effective and associated outcomes

Implementation Strategy	Total journal clubs reporting as top 3 effective	Outcomes linked to				
		**Attendance**	**Satisfaction**	**Knowledge/ skills**	**EBP culture**	**Practice**
Relate to clinical practice/ensure time to discuss application as group	6	x	x		x	x
Group prioritisation of journal club topics	6		x		x	
PICO developed as a group	3		x		x	
Multiple clinicians take turn to facilitate	2		x	x	x	
Protected time	4	x			x	
CASP tool used	3			x		
Consistent time and room booking	3	x				
Timetable of topics provided	2		x	x		
Email reminders	2	x				
Research mentor attends initially	2		x	x		
Mentor pairing	2			x		

### Relate to practice

#### Relate to clinical practice and ensure time to discuss application as group

Participants across all six journal clubs reported that relating the topic to clinical practice and ensuring enough time to apply the findings to clinical practice was a very effective implementation strategy. Relation to clinical practice influenced the EBP culture of members by increasing team discussions about integrating evidence into practice, *“it's just generated more conversation around clinical practice and providing that clinical justification of why you are or aren't doing gold standard or best practice.*” (FG 14). The strategy also enabled improved satisfaction due to increased engagement, “*I feel like that really makes me buy into journal club more too because I feel like it's not just an article, it's what are we doing clinically? Does it match up? Do we need to change our practice?…I haven't been in..a [health service] that's done that before and I like that additional step” (FG 17).* Another benefit was its importance in influencing clinical practice; *“if you haven’t had the discussion to see how it’s clinically relevant, then all you have is the theoretical knowledge”* (FG 7).

Two contexts may have influenced outcomes relating to this strategy. Firstly, journal club size was reported to impact clinician engagement in discussions about application to practice, with smaller groups finding discussion easier, “*But here I found that [journal club name] quite a small group, quite a tightknit group, so we're … open to discussion like that” (FG 4).* Secondly, multidisciplinary journal club participants found it more difficult to relate journal club topics to practice, “*it has brought challenges…I think, also in terms of topics. Finding things that are …of interest of everybody and more multi-disciplinary related”* (FG 20). This could at times have impacted attendance as clinicians would base their attendance on, *“how relevant that might be to them in their practice.” (FG 20).*

### Group participation

#### Group prioritisation of topics

Participants across all six journal clubs reported having a group-based approach to prioritising topics to be discussed in their journal club to be an effective strategy. Clinicians reported this strategy led to satisfaction with the journal club due to increased clinician buy-in and improved the journal club and resultant EBP culture, *“There was times in the past where there were certain topics that weren’t necessarily applicable to big groups of people and that really impacted, I think, the culture and maybe the attention that people paid to topics. But because now we choose topics that are relevant to the majority of [our profession], because we vote on them, that’s been really effective”* [FG 13]. This engagement also assisted in finding presenters, “*it helps with finding presenters as well because often at that time the person who's raised the topic or someone will show particular interest in it and then you can flag that person as someone who might be interested or have some extra knowledge in that topic to present on it.*” [FG 17]. A research mentor stated the strategy to prioritise topics as a group was more effective in the context of someone embedded within the team driving it rather than an external research mentor; *“the prioritisation of topics, because I’m not permanently embedded in that team and that wasn’t being driven…. I think it sort of comes back to having people—at least one person having capacity to drive and organise it* (FG 19).

#### PICO developed as a group

Participants from three of the journal clubs reported the implementation strategy of developing a PICO question (being a structured approach for framing research questions to assist with searching the literature [17]) to be effective. This strategy was reported to increase satisfaction due to the added clinical relevance it may bring, “*I think as a group it already makes sure that we are really capturing the difficulty, or really capturing what the issue at hand is”.* (FG5). Another clinician reported that the mechanism positively influenced the EBP culture, *“it has really influenced EBP within our team.”*(FG 9).

#### Multiple clinicians take turns to facilitate

Having multiple clinicians taking turns to facilitate (lead) the journal club was reported as an effective implementation strategy by two journal clubs. A number of clinician-facilitators reported that their skills as facilitators improved from having the opportunity to facilitate*, “I think personally I'm going through articles more systematically now”* (FG17), and also improved the EBP culture of the team, “*I think the more people who come through the better it will be for the general culture of EBP”. (FG13).* Another clinician commented it *assisted with more active ownership, “not taking a back-seat approach in journal club and just receiving information, but actually going and looking for it” (FG13).* Other clinicians reported having more clinician facilitators could improve satisfaction by reducing the workload on one person*, “Potentially a third could take the load off two people as well”* (FG20). Research mentors agreed, with one describing, *“I don't think one clinician can do that long term just in terms of the workload….so having other people that can rotate in that role I think [is useful].* (FG18).

#### Group-based critical appraisal

The implementation strategy of having group-based discussion as part of the critical appraisal was reported as an effective strategy by two journal clubs. Clinicians reported this helped to increase the rigour of their critical appraisal, *“we're probably doing it more rigorously when we're being questioned by other people as well and discussing it in a group. You just think about it more and talk about it more. So it's probably better analysis.”* (FG16). Satisfaction with the journal club and engagement also increased, “*group participation has been a lot better than it used to be where someone would bring a journal and they had appraised it themselves where people just sit back and listen… I think we've seen a lot more group involvement and a lot more interest”* (FG17). A clinician-facilitator agreed, *“I think that helps from a satisfaction point of view. It’s not just listening to one person talk”* (FG5).

This implementation strategy was influenced however by the size of the journal club, with larger journal clubs limiting engagement, “*quite a large department where if the question's opened up to everybody people don't want to get involved* (FG17) and also having multiple sites, in which case breaking up the discussion into smaller groups was undertaken, *“ we split up into groups and go through questions really encourages group involvement more. I think that's really positive”* (FG17).

### Consistency and structure

#### Protected time

Participants from four of the journal clubs reported having protected time in the context of the manager making attendance mandatory as being a very effective implementation strategy. This strategy was reported to facilitate attendance, *“the fact that it is compulsory means that we've got good attendance.”* (FG 16) and assisted to build the EBP culture as described by another clinician; *“it would be fantastic if they [other journal clubs] could have as much support from their managers as we do to protect that time because it promotes a culture of evidence-based practice and it’s really an expectation…once that culture has been built up its really then easy to continue it on and to continue that attendance”.* (FG 13).

#### Consistent time and room booking

Three journal clubs also reported having a consistent time and place booked as an effective implementation strategy. This was reported to facilitate attendance; *“I think definitely for attendance consistent time and place booked so that’s expected and reminders and calendar reminders that journal club is coming up that was very useful.” (FG 20).*

#### CASP tool used

Three of the journal clubs reported the use of the CASP tools as one of the most effective strategies to implementing their journal club. Participants reported that the structure of the tool helped their ability or skills in appraising articles “*I think having the CASP tools greatly helps, when looking at particular articles, and in analysing them appropriately, as much as what we can, because we’d just probably be going through the article and picking things out, whereas it gives us a bit more of a structure (FG 15)*. However, one clinician reported that their ability to understand the CASP tool was influenced by changing study designs, *“… sometimes when the study design changed, it was actually really difficult to understand what the CASP tool was asking” (FG21).* Others reported needing time to use the tool*; “sometimes we just moved, I think, quite quickly through the CASP tool…sometimes I was just slow to catch up”* [FG19].

#### Timetable of topics

Two journal clubs reported developing a timetable of topics and allocated presenters as an effective strategy. This was reported to help facilitate engagement and resultant satisfaction, as one clinician reported, *“I think the HP3s were more engaged because they knew it was coming. They would prepare in advance. They'd get their head around it more…”* (FG 14). However, this didn’t always work as planned, with a research facilitator reporting, *“I found that a lot of people wouldn’t necessarily come to me. They’d come to me about two days before it was journal club and it’s like, we’ve got to get this [article]. Then they brought in—we talked about it with the facilitators and said, look, we need to have this article and the study design sent, by email, at least two weeks before”* (FG 19).

#### Email reminders

The implementation strategy of email reminders was also reported to be an effective implementation strategy together with the article being given in advance, promoting attendance*; “Just the reminders, and warning people, I guess. Giving people the article the month before, we hoped would increase their likelihood of actually reading the article and then attending, because I think you’re less likely to attend if you haven’t had the chance to read the article”. (FG20).*

### Mentorship

#### Research mentor attends initially

Two journal clubs reported having a research mentor attend the initial journal club meetings as an effective implementation strategy. Clinicians reported that it helped facilitate their knowledge and skills, *“I think [research mentor name] was awesome coming to the journal clubs, because often we had questions that we could not answer, because some of the journal articles were that complex that we didn't really understand…she was almost able to describe it in more basic terms. That was good.”* (FG 21). Other clinicians reported it improved satisfaction and engagement, “*I think people are more prepared then because they know what questions they want to ask her and they want her opinion on things … it definitely encourages accountability [laughs]. I do think—because they know they're going to understand it by the end, which we probably can't necessarily guarantee them*.” (FG14). Clinician-facilitators also wanted the research mentor to be a more regular presence rather than just initially (FG20). Research mentors also were aware of the balance of providing support but empowering clinicians to find answers themselves as to not get too dependent, *“just making sure that we are making them think more. I had to restrain myself sometimes…which I must admit sometimes I probably did just give the answer” (FG18).*

#### Mentor pairing

The implementation strategy of pairing less experienced clinicians with more experienced as pairs to present journal clubs was reported by two journal clubs as being an effective implementation strategy. This was reported to assist with clinician’s confidence in their abilities, *“it helped them with their confidence to present because they didn't feel they were in it alone”* (FG12).

### Context-Mechanism-Outcome (CMO) configurations

Eleven CMO configurations further summarise each of the described strategies and mechanisms they enable, their resultant outcomes and influencing contexts (see Table 3), with six of these having no clear influencing contexts.

**Table 3:** CMO configurations

1. Smaller journal clubs (context) from a single profession (context) who discuss as a group the application of the journal club topic (strategy) increase team discussions and engagement about integrating evidence into practice (mechanism) resulting in increased satisfaction and attendance with the journal club (outcomes)
2. When there is someone embedded within the team to organise (context) prioritising topics for the journal club as a group (strategy) this enables increased relevance of topics and clinician buy-in (mechanism) which leads to increased satisfaction and culture (outcomes)
3. When looking at consistent study designs and going through not too quickly (context- participant experience) the structured CASP critical appraisal tool (strategy) enables repeated exposure and time to process critical appraisal principles (mechanism) which support knowledge and skills of journal club participants (outcome)
4. When breaking larger groups into smaller subgroups (context), discussion of the appraisal tool as a group (strategy) enables opportunities to share ideas (mechanism) which facilitates skills and knowledge and increases satisfaction with the journal club (outcome)
5. When managers make journal club mandatory (strategy), clinicians have protected time and expectation from managers to participate in journal club (mechanism), which can lead to improved journal club attendance and EBP culture (outcome)
CMO configurations without a clear context:
6. Having multiple clinicia- facilitators support the journal club (strategy) can provide increased opportunities for staff to practice skills, take ownership and share the responsibility (mechanism) and result in improved EBP culture, satisfaction and knowledge and skills of the clinician-facilitator (outcome)
7. Developing the PICO as a group (strategy) enables increased clinical relevance of the articles discussed and clinician buy-in (mechanism) and lead to improved satisfaction and culture (outcomes)
8. Having a consistent time and place booked and having email/calendar reminders (strategy) enables staff to more easily remember to attend (mechanisms) and facilitates journal club attendance (outcome)
9. Having a research mentor attend journal club initially (strategy) enables mentors to breakdown more complex concepts of appraisal, empower learning and enhance accountability of members (mechanism) which facilitates participant’s skills and knowledge and satisfaction with the journal club (outcome)
10. Having a timetable of topics circulated in advance (strategy) assists with preparation for and engagement in the journal club (mechanism) which can facilitate satisfaction and knowledge and skills (outcome)
11. Pairing clinicians with a mentor when presenting at the journal club (strategy) improved confidence from learning from someone more experienced rather than doing it alone (mechanism) which facilitated knowledge and skills (outcome)

### Other factors influencing outcomes

In addition to the discussed mechanisms, participants reported other factors to influence the outcomes of attendance, EBP culture, practice change and satisfaction that are inherent in the healthcare context. This included clinician workload impacting attendance, “*We are all busy, a lot of us have high caseloads or on top of it being maybe even part time that might be a challenge.”* (FG6). Staffing changes including sick leave further impacted attendance and satisfaction as one participant described, *“there’s only two people that work that day and one’s off sick and then one was on the wards and they had 15 people, people would be really cranky from the other things when they didn’t turn up for journal club, that they just couldn’t justify that bit out of the day”* (FG19). While the quality of articles appraised including their sample size power impacted on the ability to make practice changes and resultant satisfaction.

### Suggestions for future implementation from participants

An additional theme identified in the data related to suggestions for future implementation. To support knowledge, clinicians suggested having regular EBP training for all staff members as well as ongoing research mentor support and processes of handover to new staff to introduce the format. Additional suggestions for the future included the involvement of students as presenters, with a clinician suggesting, *“to actually pose the clinical questions to the students who are on placement and then for them to present during the meetings” (FG11);* running separate journal clubs within clinical areas or streams, *“I think, potentially, having articles in streams would be more beneficial looking towards the future once we're more comfortable with the whole journal and article reviewing” (FG 14).* and changes to journal club frequency ranging from fortnightly to bi-monthly. Clinicians also suggested that the implementation of journal club findings may be supported in the future by bringing action items to the team planning day or having journal club actions as an “*agenda item*” (FG5) for operational meetings.

## Discussion

The findings of this study identified which implementation strategies allied health professionals perceived to be the most effective for enhancing the outcomes of their structured TREAT journal club including attendance, culture, and satisfaction as well as contexts and factors influencing the strategies. These strategies were broadly categorised as relating to clinical practice, group participation, consistency and structure and mentoring. Drawing from elements of a realist evaluation approach allowed for the identification of Context-Mechanism-Outcome configurations as well as strategies which enabled mechanisms,to further elucidate what worked for whom and why, as well as providing a richer context to survey data obtained from earlier research [4]. Insights into specific contexts that may influence outcomes provide further practical guidance for clinicians wanting to sustain a structured journal club unique to their context.

The use of resources (such as clinician time) to implement interventions that are not sustained can be considered wasteful [18]. This study is the first to our knowledge that has explored implementation strategies for sustained journal club implementation and provides guidance on their effective implementation within health organisations. There is currently limited use of implementation science when exploring education of health professionals [19]. This study explores the effectiveness of implementation strategies, building on current evidence regarding which implementation strategies are most effective amongst allied health professionals, as highlighted in a recent systematic review which reported that research in this area is currently lacking [20].

This study revealed that a number of strategies were perceived by clinicians to facilitate positive implementation outcomes, which is consistent with previous literature in allied health that a multifaceted approach using a cluster of strategies is likely be most effective for implementation [20]. The strategies reported as effective by all six TREAT journal club related to application to clinical practice and group participation including ensuring there was enough time to discuss the clinical application as a group and having group prioritisation of journal club topics. These strategies were influenced by context, with smaller groups of single professions more likely to have enhanced outcomes from these strategies. The strategy of discussing clinical application of evidence was also reported to be most easily sustained in earlier research [10], and reported to enhance motivation. Indeed, in a time poor environment, clinicians seek out opportunities where there will be the most impact or influence on practice. Simply discussing articles which may be of interest “just in case” are reported to have less impact than a “just in time” approach whereby teams are aiming to answer a timely clinical problem relevant to the current workload [21]. Therefore, ensuring the journal club topics were of relevance and having clinicians prioritise and have input in these discussions is vital. A “just in time” strategy also aligns with principles of adult learning which state that content must be relevant and useful to the learner’s real life or in this case, everyday practice [22].

Related to the theme of consistency and structure, the strategy of “protected time” is also important as having managers that make journal club mandatory enabled increased engagement and EBP culture. In the current fiscal environment, managers may feel conflicted with taking clinicians away from clinical duties to participate in non-clinical tasks such as journal club. Indeed, during the COVID-19 pandemic, professional development opportunities were frequently cancelled [23]. The potential benefits of a strong EBP and research culture on quality of patient care and healthcare performance [24] are important but may be more subtle and incremental compared to pressing needs of patient and service demands. Managers need to weigh the longer-term potential positive impacts on culture and skill development of their team with immediate clinical needs when adopting journal clubs.

Clinicians also highlighted the value of having research mentors present in the JC to support knowledge and skill development and overall satisfaction and expressed a desire for more of this support in the future. The benefit of incidental teaching and support that a more experienced clinician-researcher or academic may offer to journal club confirms earlier findings [10]. More generally, such tailored guidance through mentors is also frequently used as a capacity building strategy to support implementation activities [25].

The study further identified that specific contexts including smaller and single profession journal clubs had potentially greater outcomes when discussing the application of the journal article to practice. Consistent with this finding, Burgess et al., describe learner’s understanding is enhanced in small groups when they can compare and build on their own knowledge collaboratively with their peers [26]. The finding of single professions reporting more positive outcomes should be interpreted cautiously as the majority of journal clubs were of a single discipline in our study. Even so, findings may align with recent qualitative research, whereby students learning with another discipline felt less motivated to learn content that did not directly apply to their healthcare practice, and regarded it as only ‘nice to know’ [27]. To maintain motivation, multidisciplinary journal clubs may therefore need to ensure articles they are discussing can be applied across professions.

### Limitations and future directions

While participants from six different journal club contexts participated in interviews, they were all from the same public hospital and health service and were all allied health professionals. Future research evaluating the sustainability of TREAT or other structured journal clubs across other geographical and clinical settings (e.g., private practice) and medical and nursing professions would be valuable to understand if similar strategies work across these contexts. Gaining perspectives from managers may also be valuable. The timing of the current research meant that due to COVID-19 impacts and increased staffing pressures two of the journal clubs could not participate in the 16-month evaluation. A minimal realist approach was used to deepen understanding of where and why implementation strategies were considered to be effective. The way in which contextual factors influenced key implementation strategies to achieve specific outcomes may be helpful in applying similar implementation strategies across a range of different organisational contexts. Further theoretical investigation is recommended to confirm the way these contextual factors influenced implementation strategies and respective outcomes. While our conceptualisation of implementation strategies as enablers of mechanisms provided useful information about how these implementation strategies worked in different circumstances, future research could explore the deeper generative mechanisms and behavioural processes underlying these strategies to provide a more robust realist evaluation.

### Implications

The present research distilled key implementation strategies for sustaining TREAT journal clubs that may be useful for services to consider for their contexts when implementing journal clubs, as well as providing some practical insights into what works for whom and in what contexts. Health professionals may choose to consider four main elements to support effective journal club implementation including: relation to practice, consistency and structure, mentorship and group participation. More specifically, working through processes of the journal club as a group including discussion of clinical application as a group, group prioritisation and developing the PICO as a group are important to actively engage all members in the journal club process. Having the structure of a consistent time and place that is endorsed by leadership (ideally giving mandatory protected time) and the use of structured critical appraisal tools (e.g., CASP tool) are also important to enhance journal club attendance and outcomes. A team approach and mentorship including sharing the role of facilitator across multiple clinicians, mentor pairing and including a research mentor may further support knowledge and skills and overall satisfaction. Participants also suggested integrating regular EBP training and processes for introducing the TREAT format to new staff, which although was not explored as a strategy in this research may be useful for ongoing sustainability.

Contextual findings indicated that smaller and single profession journal clubs had potentially greater outcomes when implementing the strategy relating to discussing the application of articles to practice. Clinicians wishing to establish a journal club should therefore consider these contextual factors where possible when composing a club. For existing larger journal clubs, it is recommended to break into smaller groups during the session to facilitate participation and discussion regarding clinical application. For multidisciplinary and larger journal clubs thoughtful consideration must also be given to ensure that topics are relevant across members. If such topics cannot be identified, the benefit of participation in informing practice may not be worth the opportunity cost to the clinician in terms of time taken from other clinical priorities. Alternatively, if fewer topics can be identified that are relevant across members, the team may want to consider reducing the frequency of their meetings to ensure they are only meeting to discuss topics that are meaningful to all members.

## Conclusion

The present research identified several strategies which may facilitate enhanced journal club attendance and satisfaction, EBP culture and knowledge and confidence of clinicians when implementing a TREAT journal club long term. These strategies may be helpful for organisations and clinicians to consider when wanting to sustain a journal club within their own setting. While specific influencing contexts were identified including journal club size and whether it was multi or uni-disciplinary, further research across broader settings and professional groups would be beneficial to further explore the influencing contextual factors using a more theoretically informed approach.

## Supplementary Information

Supplementary Material 1.

Supplementary Material 2.

## Data Availability

Deidentified data is available on request.
