# Supplementary material for: What do clinicians perceive as the effective implementation strategies for TREAT journal clubs? A qualitative study
Source: BMC Med Educ. 2025 Mar 24;25:427. doi: 10.1186/s12909-025-06929-x (PMC11931826; doi:10.1186/s12909-025-06929-x)
Supplement: Supplementary file 1 — Supplementary Material 1. [file 12909_2025_6929_MOESM1_ESM.docx]

# Supplementary file 1:

# General session guide: TREAT format

| Activity | Detail | Time |
| --- | --- | --- |
| **Assign Scribe**  **Take roll** | Introduce self and roles (first session only) Nominate scribe for the session.  Hand around roll for people to sign. | **5min** |
| **Review actions from last meeting** | Follow up previous actions from last meeting and troubleshoot any barriers. Modify new actions if needed (documented by scribe). |  |
| **Clinical Question** | Clinical question/background introduced by presenting clinician(s) | **5min** |
|  | Process of forming PICO and search strategy (database, terms, limiters) for identifying article discussed with presenting clinician |  |
| **Abstract reading & Appraisal**  **(CASP)** | **Everyone reads the abstract** independently (2min to a though)  Complete **first two questions of Section A together** as group and decide whether worth continuing appraisal  **Rest of Section A then appraised as a group**. For larger groups or to increase involvement, break up into groups of 2-3 and look at different questions of the tool (e.g., pairs do 1 questions each and then come back to discuss after 5-10 minutes).  **Discuss Sections B together** | **25 min** |
| **Application to context** | **Discuss section C together** (how relates to current clinical context, patient values, experience etc), How does this influence current practice? | **15 min** |
| **Actions/ Follow up** | Discuss any follow up required regarding implementation of evidence and who is accountable (to be followed up at subsequent session) which is documented by the scribe.  Review topic and presenter for next meeting (brainstorm topic if none allocated). | **5min** |
