# Supplementary material for: What do clinicians perceive as the effective implementation strategies for TREAT journal clubs? A qualitative study
Source: BMC Med Educ. 2025 Mar 24;25:427. doi: 10.1186/s12909-025-06929-x (PMC11931826; doi:10.1186/s12909-025-06929-x)
Supplement: Supplementary file 2 — Supplementary Material 2. [file 12909_2025_6929_MOESM2_ESM.docx]

**Supplementary file 2: Interview Guide**

**Clinician 10-Month Focus Group Interview Guide**

1. How would you describe your experience with participating in the TREAT journal club format over the last 10 months?
2. Were there any challenges you faced with sustaining the journal club? Please describe.

What strategies did you use to try to overcome these?

How would you describe their impact? (did that strategy work/why?)

At the beginning of the trial, some specific strategies were chosen to assist with implementing the journal club. We are now going to discuss how useful each of these strategies were. *Interviewer goes through each implementation strategy originally identified by team*

Which 2-3 strategies, if any, that you feel had the most positive impact on your journal club? (e.g., impact on attendance, culture, satisfaction). Why do you think these strategies had a positive impact?

*Which 2-3 strategies, if any, do you feel had the least impact on your journal club?
Why do you think these strategies had little impact?*

1. What other factors (if any) do you think may have influenced clinician’s attendance?

- Where there any other strategies you used to increase attendance?
- If so, were these effective? Why/Why not.

1. What factors (if any) have influenced the EBP culture within your team over the last 10 months?

- Where there any other strategies you used to enhance EBP culture?
- If so, were these effective? Why/why not.

1. Were there changes in practice (clinical or service) which have occurred as a result of your journal club?

- If so, can you give examples of this?
- What helped or hindered this practice change from happening?

1. Have you observed changes in individual knowledge and/or skills ?

If so, what strategies may have assisted this?

1. Did you observed sharing or learning within your team during the journal club?

If so, what assisted/hindered this?

1. What does your journal club plan to do in the next 6 months?
2. Do you have any other comments regarding implementing the journal club or suggestions for others potentially implementing a journal club.

**Clinician 16-Month Focus Group Interview Guide**

1. How would you describe your experience with participating in the TREAT journal club format over the last 16 months?
2. Were there any challenges you faced with sustaining the journal club, particularly over the last 6 months/6 journal club sessions? Please describe.

What strategies did you use to try to overcome these?

How would you describe their impact? (did that strategy work/why?)

At the beginning of the trial, some specific strategies were chosen to assist with implementing the journal club (facilitator refers participants to the original implementation plans).

Are there any new strategies you have implemented in the last 6 months to support running the journal club not listed here?

We are now going to discuss how useful each of these strategies were. *Interviewer goes through each implementation strategy originally identified by team/ and new ones.*

Which 2-3 strategies, if any (they can be new or existing strategies), that you feel had the most positive impact on your journal club? (e.g., impact on attendance, culture, satisfaction). Why do you think these strategies had a positive impact?

*Which 2-3 strategies, if any, do you feel had the least impact on your journal club?
Why do you think these strategies had little impact?*

1. What other factors (if any) do you think may have influenced clinician’s attendance?

- Where there any other strategies you used to increase attendance?
- If so, were these effective? Why/Why not.

1. What factors (if any) have influenced the EBP culture within your team over the last 16 months?

- Where there any other strategies you used to enhance EBP culture?
- If so, were these effective? Why/why not.

1. Were there changes in practice (clinical or service) which have occurred as a result of your journal club?

- If so, can you give examples of this?
- What helped or hindered this practice change from happening?

1. Have you observed changes in individual knowledge and/or skills ?

If so, what strategies may have assisted this?

1. Did you observed sharing or learning within your team during the journal club?

If so, what assisted/hindered this?

1. What does your journal club plan to do now?
2. Do you have any other comments regarding implementing the journal club or suggestions for others potentially implementing a journal club.

**Example Interview Guide Research Facilitators**

**These are example questions only.**

1. How would you describe your experience with facilitating the TREAT journal club format as part of the trial?
2. Were there any challenges you faced with helping facilitate the journal club? Please describe.

What strategies did you use to try to overcome these?

How would you describe their impact?

At the beginning of the trial, some specific strategies were chosen to assist with implementing the journal club *[facilitators refer to implementation plans developed with clinicians].* What do you think were the top two most useful strategies you observed in the journal club you helped facilitate and why?.

What do you think were the two least most useful strategies and why?

1. How would you describe your role in supporting building the internal capacity of clinicians to facilitate the journal club?

Where there any things you did which helped facilitate their development? Provide examples.

What did you find most challenging?

1. Have you observed changes in team’s knowledge and/or skills?

Can you provide examples?
If so, what strategies may have assisted this?

1. How would you describe the sense of ownership that the clinicians had of the journal club?

What do you think helped this?

What do you think was challenging?

1. Did you observe sharing or learning between team members during the journal club?

If so, what assisted/hindered this?

1. What other factors (if any) do you think may have influenced clinician’s attendance?
2. What factors (if any) have influenced the EBP culture within the team over the last 10 months?

- Where there any other strategies you used to enhance EBP culture?
- If so, were these effective? Why/why not.

1. Do you have any suggestions or modifications to the current TREAT journal club format to support its implementation?
2. What do you think would be the best way for facilitators to learn about implementing the TREAT journal club format with teams?
3. What advice would you give to another research facilitator who was considering helping facilitate a journal club with clinicians?
